# On Irregular Malarious Diseases, and Their Counteraction by Means of Strychnine

**Published:** 1849-10

**Authors:** 


### ECLECTIC DEPARTMENT,

### AND SPIRIT OF THE MEDICAL PERIODICAL PRESS.

---

*On Irregular Malarious Diseases, and their Counteraction by means of Strychnine.* By J. P. Kirtland, M. D., Prof. Phys. Diag. and Theo. and Prac. of Med., Cleveland Medical College.

Malaria has produced the larger share of fevers that have prevailed in Ohio. Those of a continued form, originating from other causes, have occasionally intercurred, and have not, in all instances, escaped the modifying influences of that agent.

All varieties and species of acute febrile disorders have varied in the quality of their action, in obeyance to the diathesis or epidemic constitution, which, during the last fifty years, has developed in this State, every grade, from the highest sthenic to the lowest asthenic.

Malaria has also induced many obscure and anomalous forms of disease, and frequently imparted new features and tendencies to every disorder that may come within the sphere of its influence.

To these irregular manifestations of its powers, and to certain means adapted to their counteraction, we would respectfully invite your attention.

That it makes its impressions, primarily, on the *cerebro spinal system*, can hardly admit of a doubt. A portion of that system is, as a consequence, impaired, which portion may be the base of an important nerve or nerves, distributed to various organs and structures. The morbid impressions may be transmitted to the extremities of these nerves, and there develop its evidences, in the form of some functional or organic derangement.

The viscera of the chest, abdomen and pelvis, and the fibrous cellular tissues may, in this way, become the seat of disease, from reflected malarious impressions. Such cases are common. Many affections of the heart, stomach, intestines and liver, and, in females, uterine disorders, are of this character.

They have been imperfectly recognized, and described by authors as "Irregular and masked intermittents," "Complications," &c. This exciting cause may restrict its action solely to the nervous system, and has been known to originate or complicate with every species embraced in Cullen's class of "Neuroses," from Apoplexy down to Hysteria.

In other instances it may take a wider range, and show its effects under the forms of irritation, inflammation, and I might with propriety add, every disorder to which the human family is subject.

In all cases of malignant erysipelas, that have come within my experience, it has participated largely, either as an exciting or modifying cause.

To the western physician, under whose observation it is daily producing such effects, their diagnosis is often a source of perplexity; while to our eastern medical brethren, who know nothing of such diseases, except from books and lectures, it is a perfect stumbling block.

Nothing short of an intimate acquaintance, gained by experience and correct observation, can enable the practitioner to detect and comprehend the insidious and ever varying forms of malarious action.

They generally show some tendency to periodical attacks, but it may be so obscure and irregular as to escape the attention of a superficial observer. The circumstance of a patient having been exposed to this agent may occasionally aid in detecting the character.

If the *diagnosis* be correctly formed, the *prognosis* will, in most instances, be favorable.

On the other hand, if their intimate nature be overlooked, and attention directed merely to their more apparent symptoms, improper medicines will probably be prescribed that may induce more disorder than they will correct.

Even in cases that present a fair prospect of an ultimate cure, it may not be possible to suddenly remove the malarious impressions from the nervous centres, especially if strengthened by time and habit. They are apt to remain for a long time, developing their effects without regard to weather, season and ordinary medication.

A frequent consequence is the expenditure of vitality and reduction of the system to a condition in which tuberculous matter will be deposited in the liver and lungs.

Prior to 1827 the only form of Phthisis known on the Connecticut Western Reserve, was of this secondary character.

*The indications of cure,* which naturally present themselves, are:

1. To *counteract* malarious action.
2. To *correct* any morbid change that may have occurred. Our remarks will be confined to the first indication.

Experience has demonstrated that under our present diathesis, in northern Ohio, *reducing* agents, *used as such,* are not appropriate means for curing these forms of malarious disease.

The lancet, emetics and cathartics, may occasionally be required *to change* the action, but not *to reduce* the system—a distinction, both correct and important, not always observed, either in practice or medical writings.

Specifics have not yet been found; bark and its preparations, even the valuable alkaloid, quinia, have all sometimes failed to effect a complete and permanent cure.

Capsicum, opium, preparations of iron, stramonium, wine, ale, brandy, hydriodate of potash, &c., have been frequently tried, singly and in different combinations, with only partial success.

Fowler's solution and tincture of opium, combined, have formed a favorite remedy with me, until recently. With all these means of counteraction at command, the result of the treatment has not, as a whole, been satisfactory.

On attempting to effect a cure it would be philosophical to select agents that would determine their action to the original seat of disease. The woodman would not commence lopping off the extremities of the limbs for the purpose of falling the tree, but would direct his blows to the main trunk.

If the view we have taken of the original seat of malarious impression be correct, it follows that the pathological condition we are required to treat, is an *impairment of the nervous centres, the evidences of which are exhibited in certain trains of symptoms of the nervous extremities.*

Our object would, of course, be to remove the morbid impression from whence those trains arise, and not direct our attention to the extremities where more symptoms are developed. The mode of accomplishing it is to excite in the nervous centres a new and artificial action, more compatible with a healthy condition than is the disease, and to carry it so far as to counteract and overcome the original morbid action.

Medication may then be withdrawn and health will resume her province.

On theoretical grounds we should look upon strychnine as well adapted to this purpose, as its powers are exerted principally on the cerebro-spinal system, when it excites an action of its own kind.

Experience has tested and established the correctness of this view. Some ten years since we prescribed it in many cases in the Commercial Hospital, at Cincinnati, with favorable results, except, from inattention on the part of those having immediate charge of the sick, the remedy was allowed to be given in too heavy doses, and it produced unpleasant factitious symptoms in a few cases.

We are, however, indebted to my colleague, Prof. Ackley, for its recent introduction into practice in northern Ohio, on more definite principles. It is now extensively employed by several intelligent physicians, and volumes of evidence in its favor might, if necessary, be laid before you.

Some cautions are required to regulate its use. If it be urged with too much rapidity, or continued beyond the point at which it begins to manifest evidence of its having established its control over the nervous system, factitious symptoms may arise that will either retard or defeat the cure.

Increase of sensibility and tenderness along the spine, particularly along the scapulæ, should warn us to withhold the remedy for a time. Carried beyond this point it will induce swelling, stiffness and rheumatic condition of the joints. Severe neuralgic pains, spasms of the diaphragm, ringing in the ears, and general nervous irritability. It is necessary to allow its effects to entirely subside during the interval of its discontinuance. After one, two, or three weeks, the course may be repeated, and again, from time to time, as occasion may require. A failure to effect a cure by the first trial, should not discourage its repetition.

In many cases it may be relied on solely; in others it may be necessary to precede or accompany it with certain adjuvants, as mercurials and quinine. A combination with the latter will often prove more effective than either of the articles employed separately. To what extent it may be substituted for quinine, in the treatment of regular intermittents, remains to be decided by further experience. That it will answer the purpose in some cases, and fail in others, I have ample evidence.

A malarious action counteracted by it, is less liable to recur than when that purpose has been accomplished by quinine.

It is an important query, whether it may not be employed at a certain juncture in the forming stage of remittent fevers, and, perhaps, during the first part of the active stage, to counteract the malarious impression, and arrest its further progress. The subsequent symptoms that usually arise, are mostly the results of the impairment of the cerebro-spinal system, by such impression.

Many cases of our remittent fevers, as well as the malignant remittents of the south, have of late years been arrested at those stages, by means of a prompt use of quinine.

Much will depend on the form and mode in which strychnine is employed. In substance its effects are not always uniform and certain. The amorphous powders are impure, but the crystals are incapable of adulteration. The solution is the most preferable form for its use.

Through the kindness of Mr. Hall, of the firm of Fiske & Hall, an intelligent and experienced druggist of Cleveland, I am enabled to lay before you the formula for "Hall's Solution of Strychnine."

℞. Strychnine crystals, xvi. grs.
Water.
Alcohol, aa ℥ viiss.
Acetic Acid.
Tinct. Cardamom comp. aa ℥ ss.
M. F. Sol.

Dose 20 to 30 drops, three times each day.

One drachm of the solution contains 1-8 of a grain of strychnia. Adults will bear that amount for a dose, yet in ordinary cases it is better to make our approaches slowly, 1-32d to 1-16th part of a grain may be sufficient. Certain empirics are using it in doses of 1-8 of a grain, and calling their practice "Homœopathic."

For the purpose of illustrating its powers and efficacy, permit me to state that I have been familiar with a case that originated from repeated exposures to malaria. It commenced with muco-enteritis, that at length terminated in dyspepsia, irregular and vitiated secretions from the liver, constipation, neuralgic pains in the bowels and limbs, and at 11 o'clock each day, a paroxysm of tic doloreux of the left supra orbita nerve, that was followed with an ichorous discharge into the nostrils from the sinuses of the affected side. Medication and circumstances would occasionally interrupt the violence of the disease, but it was never, during fourteen years, entirely absent.

Two years since the derangement of the liver and constipation became unmanageable, and resulted in a series of attacks of colic and peritoneal inflammation, and at last in a fully developed intermittent fever.

This latter phase was arrested by means of quinine, but the other prominent disorders continued. Last February the patient, in a reduced and suffering condition, was put upon the use of strychnine, *without any other medication whatever.*

At the end of four days evidences were apparent that the nervous centres were aroused, and their action transmitted to the nervous extremities.

The liver began to pour out freely secretions of a healthy character, the appetite improved, the peristaltic motion became active, and lavements and cathartics were dispensed with, though for two years previous they had been in steady requirement.

The nervous pains and tic doloreux subsided, and at this time, perfect health is restored. Three different trials with the strychnine were requisite to effect this important result.

During the exhibition of the first, the patient attempted to carry into practice the common maxim that "We cannot have too much of a good thing," and in the end demonstrated to his own satisfaction, *that we could have enough.*

Finding that his case daily improved under the use of 25 drop doses of the solution, he augmented them to 60 drops.

At length his limbs were attacked with severe neuralgic pains; the joints swelled; the spinal column became sensitive and painful upon the least motion; palpitation of the heart, ringing in the ears, and general nervous irritability, and occasional spasms of the muscles of the back, ensued before he relinquished his experiment. These symptoms gradually subsided, after the cause was withdrawn.

Time will not allow me to narrate an account of its favorable effects in one of the most obstinate forms of disease that we frequently meet at the west—constipation in sedentary and nervous individuals.

The true pathology is overlooked, and the organs of digestion generally charged with being in fault. Remedies are usually prescribed that in the end only aggravate the evil.

The origin of the disease is in the nervous centres, from whence its effects are reflected to the excito-motary nerves, which govern the outlets of the system.

In this section of the country, all such cases are either produced or modified by malaria.

The remedy is Strychnine.—*Proceedings of the Ohio Medical Convention.*

